# Evaluating fermentation quality, *in vitro* digestibility and aerobic stability of a total mixed ration ensiled with different additives on Tibet plateau

**DOI:** 10.5713/ajas.19.0972

**Published:** 2020-04-12

**Authors:** Zhihao Dong, Siran Wang, Jie Zhao, Junfeng Li, Qinhua Liu, Yuhong Bao, Tao Shao

**Affiliations:** 1 Institute of Ensiling and Processing of Grass, College of Agro-grassland Science, Nanjing Agricultural University, Weigang 1, Nanjing 210095, China; 2 Institute of Grassland Science, Tibet Academy of Agricultural and Animal Husbandry Sciences, Lhasa 850000, China

**Keywords:** Additive, Aerobic Stability, Fermentation Quality, Ensiled Total Mixed Ration, Tibet Plateau

## Abstract

**Objective:**

To investigate the improvement in utilization efficiency of total mixed ration (TMR) on Tibetan plateau, TMR were ensiled with different additives.

**Methods:**

A total of 150 experimental silos were prepared in a completely randomized design to evaluate the six treatments: i) control (without additive), ii) *Lactobacillus buchneri* (*L. buchneri*), iii) acetic acid, iv) propionic acid, v) 1,2-propanediol; and vi) 1-propanol. After 90 days of ensiling, silos were opened for fermentation quality and *in vitro* analysis, and then subjected to an aerobic stability test for 14 days.

**Results:**

Treating with *L. buchneri*, acetic acid, 1,2-propanediol and 1-propanol decreased propionic acid contents and yeast number, whereas increased (p<0.05) pH, acetic acid and ethanol contents in the fermented TMR. Despite increased dry matter (DM) loss in the TMRs treated with 1,2-propanediol and 1-pronanol, additives did not affect (p>0.05) all *in vitro* parameters including gas production at 24 h (GP_24_), GP rate constant, potential GP, *in vitro* DM digestibility and *in vitro* neutral detergent fibre digestibility. All additives improved the aerobic stability of ensiled TMR to different extents. Specially, aerobic stability of the ensiled TMR were substantially improved by *L. buchneri*, acetic acid, 1,2-propanediol, and 1-propanol, indicated by stable pH and lactic acid content during the aerobic stability test.

**Conclusion:**

*L. buchneri*, acetic acid, 1,2-propanediol, and 1-propanol had no adverse effect on *in vitro* digestibility, while ensiling TMR with the additives produced more acetic acid and ethanol, subsequently resulting in improvement of aerobic stability. There is a potential for some fermentation boosting additives to enhance aerobic stability of fermented TMR on Tibetan plateau.

## INTRODUCTION

Fermented total mixed ration (TMR) has attracted attention in Tibet in the past decade because ensiling TMR can reduce daily labor for feed preparation, improve longevity, and ease long-distance transportation compared with regular TMR. Furthermore, a large number of agricultural products, such as hulless barley straw (HBS) and wet hulless-barley distillers’ grains (WHDG), are available in Tibet. The locally available HBS or WHDS may improve the characteristics of ensiled TMR and replace traditional TMR ingredients which are limited in supply. Pastoral areas are scarce in Tibet and long-distance transportation of feed ingredients are necessary. During the transportation, ensiled feeds are inevitably exposed to air, which easily leads to aerobic deterioration. Identification of effective ways to improve the aerobic stability of fermented TMR is required in the region.

Various additives have been developed to enhance the aerobic stability of silages. Inoculation with *Lactobacillus buchneri* (*L. buchneri*) is effective for enhancing the aerobic stability of silages [1]. Short-chain fatty acids, such as acetic and propionic acids, are able to suppress the growth of yeast and aerobic microorganisms, and also are commonly used as additives to prevent aerobic deterioration of silages [2]. However, effectiveness of an additive on fermentation quality and aerobic stability of silage varies depending on the materials to be ensiled. Studies on evaluating the effectiveness of the additives on locally produced TMR on Tibetan plateau are limited. Additionally, it has been reported that *L. buchneri* improves aerobic stability by fermenting lactic acid to acetic acid and 1,2-propanediol [3]. Despite acetic acid being generally considered as the main active ingredient responsible for the increased aerobic stability of *L. buchneri*-inoculated silage, some evidence suggests that accumulations of 1,2-propanediol and 1-propanol during fermentation may also be involved in the resistance to aerobic deterioration [4,5]. To our knowledge, previous studies focused exclusively on the evaluation of acetic and propionic acids, with little attention being paid to these alcohols.

The objective of this study was to evaluate the effects of *L. buchneri* and two short-chain fatty acids as well as two alcohols on the fermentation quality, *in vitro* digestibility and aerobic stability of an ensiled TMR prepared in Tibet.

## MATERIALS AND METHODS

### Total mixed ration silage preparation

Alfalfa (*Medicago sativa* L.) and tall fescue (*Festuca arundinacea Schreb*.) were cultivated in the experimental field of Rikaze Grassland Station (29° 16′ latitude N, 88° 53′ longitude E, 3,836 m above sea level, Tibet, China). The HBS was the residue remaining after harvesting grain. Alfalfa was harvested at 75% bloom and tall fescue was harvested at the boot stage. The forages and HBS were chopped to the length of 2 to 3 cm with a manual forage chopper. The WHDG was obtained from a private barley wine processing company at Rikaze, and the mixed concentrates (7.5% crack corn, 20% rape cake meal, 20% cotton seed, 27.5% distillers dried grains with soluble, 20% wheat bran, 5% vitamin–mineral) were obtained from a private small-scale dairy farm in Rikaze, Tibet, China. TMRs (640 g) were ensiled using a plastic laboratory silo (1 L capacity). The chemical compositions of all used materials are shown in Table 1, and ingredients and chemical compositions of TMR are shown in Table 2. A total of 150 experimental silos (6 treatments×5 time points×5 replicates per treatment) were prepared in a completely randomized design to evaluate the following treatments: i) control (without additive), ii) *L. buchneri*, iii) acetic acid, iv) propionic acid, v) 1,2-propanediol, and vi) 1-propanol. The *L. buchneri* was supplied by Institute of Forage Ensiling and Processing of Nanjing Agricultural University and applied at 1×10^6^ colony-forming units (cfu)/g based on fresh weight (FW) [6]. The application rates of acetic and propionic acids were 0.3% FW (equals to 6.2 g/kg dry matter [DM]) [6]. The 1-propanol and 1,2-propanediol were applied at 0.5% FW. All chemicals used were of analytical grade. The prepared silos were stored at ambient temperature, opened after 90 days and then subjected to an aerobic stability test for 14 days.

### Chemical and microbial analysis

Fresh forages, pre-ensiled TMR and fermented TMR were analyzed for chemical and microbiological compositions. Approximately 200 g of sample was oven-dried at 60°C for 48 h to determine DM content and then ground to pass 1-mm screen with laboratory knife mills (FW100, Taisite Instrument Co., Ltd., Tianjin, China) for other chemical composition analysis. Total nitrogen (TN, 978.04), ether extract (EE, 920.39), and crude ash (Ash, 942.05) were measured according to the methods of Association of Official Analytical Chemists [7]. Crude protein (CP) was calculated as TN×6.25. Water soluble carbohydrates (WSC) was determined by colorimetric after reaction with anthrone reagent [2]. The contents of neutral detergent fibre (aNDFom) and acid detergent fibre (ADFom) were measured by the procedures of Van Soest et al [8], the heat stable amylase and sodium sulphite were used for NDF procedure.

For microbiological composition analysis, 10 grams of sample was blended with 90 mL of sterilized water, and serially diluted in sterilized water. The lactic acid bacteria (LAB) was counted on de Man, Rogosa, and Sharpe agar medium, incubated in an anaerobic incubator at 30°C for 2 days. Yeasts and aerobic bacteria were enumerated on potato dextrose agar (Sinopharm Chemical Reagent Co., Ltd., Shanghai, China) and nutrient agar (Shanghai Sincere Biochemical Technology Co., Ltd., Shanghai, China) under aerobic conditions.

About 35 grams of sample was blended with 60 mL distilled water and macerated for 24 h at 4°C. The extract was filtered through 2 layers of cheesecloth and a filter paper (Xinhua Co, Ltd., Hangzhou, China). The filtrate was used for pH, organic acids and ammonia nitrogen (NH_3_-N) determinations. The pH was measured with a HANNA HI 2221 pH meter (Hanna Instruments Italia Srl, Villafranca Padovana, Italy). The NH_3_-N was determined using the phenol-hypochlorite reaction method [9]. Buffering capacity was determined according to the method of Chen et al [10]. The organic acids (including lactic, acetic, and propionic acids) and alcohols (including ethanol, 1,2-propanediol and 1-propanol) were quantified using an Agilent 1260 HPLC system equipped with a refractive index detector (Carbomix H-NP5 column, 2.5 mM H_2_SO_4_, 0.5 mL/min).

### *In vitro* incubation and dry matter degradability measurements

*In vitro* fermentation was conducted in serum bottles following the method of Contreras-Govea et al [11] with some modifications. Briefly, approximately 1 g of ground sample was placed in 130-mL serum bottles. The rumen fluid was obtained through a rumen fistula before morning feeding from four dry Boer goats fed with diet consisting of 6% alfalfa, 59% guinea grass, and 35% concentrate at 1.3 times of the maintenance level. Rumen fluid was filtered through 4 layers of gauze and mixed in the ratio of 1:2 (v/v) with buffer, and 60 mL of the mixture was transferred into each serum bottle. Each serum bottle was flushed with CO_2_ and kept in a water bath at 39°C, after being capped with a butyl rubber stopper and sealed with an aluminum crimp. Gas production was measured at 4, 8, 12, 24, 48, and 72 h using a pressure transducer technique and corrected with blank bottles. After 72 h of incubation, undigested solids were precipitated by centrifugation at 1,000 g for 10 min at room temperature, dried in an aerated oven at 65°C for 48 h and then assayed for DM and aNDF. The *in vitro* digestibility of DM (IVDMD) and NDF (IVNDFD) were calculated based on the differences in their respective weight before and after incubation.

Cumulative gas production (GP) data were fitted to the exponential equation: y = b(1−e^−ct^), where y is the volume of gas produced at time t, b is the GP from the insoluble fraction (mL), c is the GP rate constant, t is the incubation time (h).

### Aerobic stability test

After 90 days of ensiling, fermented TMR from each silo was taken out, fully mixed and loosely placed into a bigger 15 L open-top and sterile polyethylene bottle. Each bottle was covered with a double layer of gauze and stored at ambient temperature (24°C to 27°C). During the test, TMR were sampled for pH, organic acids, NH_3_-N, WSC, and microbes count analyses at 0, 3, 6, 9, and 14 days. Aerobic stability is defined as a rise in pH value of TMR by 0.5 unit above the initial pH value at silos opening [12].

### Statistical analyses

Analysis of variance (ANOVA) was performed using the general linear model procedure of SAS rev. 9.2. The data related to fermentation variables were subjected to one-way ANOVA, with fixed effect of treatments. While data related to chemical and microbial composition during aerobic exposure were analyzed using the following model: *Y_ij_* = *μ*+*S_i_*+ *A_j_*+*S_i_*×*A_j_*+*ɛ*, where: *Y_ij_* = the response variable; *S_i_* = treatment; *A_j_* = aerobic exposure; *S_i_*×*A_j_* = treatment×aerobic exposure; *ɛ* = random errors. Duncan’s multiple range test was used to separate means when significant effects (p<0.05) were detected.

## RESULTS

### Characteristics of feed ingredients and total mixed ration before ensiling

As shown in Table 1, two roughages contained similar ADF, ash, and EE. Compared with tall fescue, alfalfa was lower in DM, WSC, and NDF contents, while higher in CP and buffering capacity. The chemical and microbial compositions of the TMR before ensiling are presented in Table 2. The DM, NDF, CP, and WSC contents of TMR were 485, 418, 221, and 90.5 g/kg DM, respectively. The LAB, aerobic bacteria and yeast counts were 5.37, 6.44, and 5.21 log_10_ cfu/g FW, respectively.

### Fermentation quality of ensiled total mixed ration

The fermentation quality and microbial composition of TMR after 90 days of ensiling are given in Table 3. Additives affected all fermentation parameters (p<0.05), except for butyric acid contents and LAB number. Treating *L. buchneri*, acetic acid decreased propionic acid contents, whereas increased (p< 0.05) pH, acetic acid and ethanol contents. Adding 1,2-propanediol and 1-propanol decreased (p<0.05) lactic acid and propionic acid contents, whereas increased pH, acetic acid, ethanol and NH_3_-N contents. The 1-propanol and 1,2-propanediol were only accumulated greatly in their respectively treated TMR. Addition of acetic acid reduced (p<0.05) the aerobic bacteria counts. Compared with other TMR, the numbers of yeasts were much lower (p<0.05) in TMR ensiled with *L. buchneri*, acetic acid, 1,2-propanediol, and 1-propanol.

### Chemical compositions and *in vitro* degradability of ensiled total mixed ration

**Figure 1:**
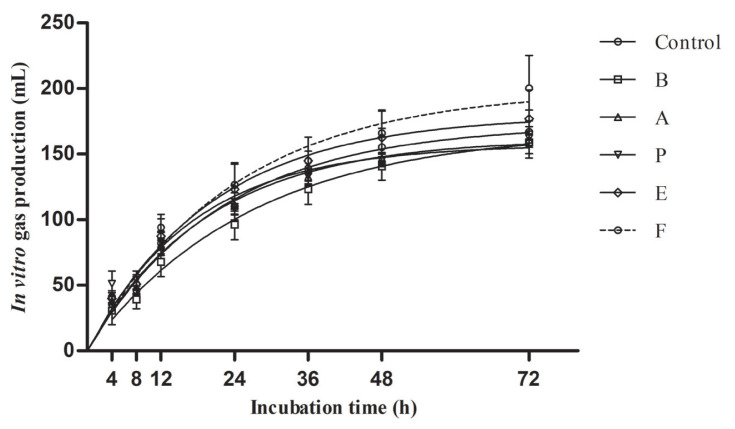
Gas production kinetics (mL/g dry matter) from *in vitro* fermentation of fermented total mixed ration for 72 h (n = 5, bars indicate standard error of the means). B, *L. buchneri*; A, acetic acid; P, propionic acid; E, 1,2-propanediol; F, 1-propanol. Gas production data were fitted to the exponential model: y = b(1−e^−ct^); the parameters b and c were estimated by an iterative least squares procedure using the NLIN procedures of SAS. There were no significant differences in gas production between the treatments during incubation.

The chemical compositions of ensiled TMR after 90 days of ensiling is shown in Table 4. With respect to chemical compositions, only DM was influenced by the additives (p<0.05). Compared with control, TMR ensiled with 1,2-propanediol and 1-propanol showed lower (p<0.05) DM contents and higher (p<0.05) DM loss. The measured or estimated *in vitro* parameters are presented in Table 5 and Figure 1. The potential GP ranged from 158 to 200 mL/g DM. Additives did not affect *in vitro* parameters including GP_24_, GP rate constant, potential GP, IVDMD, and IVNDFD.

### Aerobic stability of ensiled total mixed ration

The changes in fermentative characteristics and microbial compositions of ensiled TMR during aerobic exposure are given in Table 6 and 7, respectively. The control began to spoil after 6 days of aerobic exposure, with rises in pH and declines in lactic acid contents. All additives improved the aerobic stability of ensiled TMR to different extents. Of the additives, *L. buchneri*, acetic acid, 1,2-propanediol, and 1-propanol had superior abilities to propionic acid at improving aerobic stability, indicated by stable pH and lactic acid content. Throughout the aerobic stability test, acetic acid, and ethanol contents were always greater (p<0.05) in *L. buchneri*, acetic acid, 1,2-propanediol, and 1-propanol-treated TMR relative to other TMR. Table 7 shows the changes in chemical compositions of ensiled TMR during aerobic exposure. The DM, WSC, and NH_3_-N contents fluctuated during the test period and did not differ among the TMRs at most intervals of the aerobic stability test. The changes in microbial composition of ensiled TMR during aerobic exposure are displayed in Table 8. TMR ensiled with *L. buchneri*, acetic acid, 1,2-propanediol, and 1-propanol showed numerically, or significantly lower yeast and aerobic bacteria counts than other TMR during the aerobic exposure.

## DISCUSSION

### Additives on fermentative parameters

The fermentation quality of silage depends on the chemical and microbial properties of the material to be ensiled. To ensure satisfactory silage quality (i.e., extensive lactic fermentation and high recovery of DM and energy), a feedstuff must have adequate WSC content (>60 g/kg DM) and population of LAB (>5.00 log_10_ cfu/g FW) as well as a proper DM (300 to 400 g/kg DM) [13]. These criteria were almost met in the TMR at the time of ensiling, despite the DM being slightly higher than the normal. After 90 days of ensiling, the butyric acid was undetected and lactic acid was dominant among the fermentation products in all ensiled TMRs (Table 3). Treating with *L. buchneri* and acetic acid increased pH, acetic acid, and ethanol production in the TMRs. *L. buchneri* is well known as a heterofermentative LAB species. Altered fermentation in *L. buchneri*-inoculated silage might be attributed to the increased dominance of *L. buchneri* in epiphytic microflora that shifted the metabolism to a more heterofermentative process, while that in acetic acid-treated silage might be related to the depression of homofermentative LAB species considering that heterofermentative LAB are more tolerant to acetic acid than homofermentative LAB [14]. Similarly, Ren et al [15] also found that addition of acetic acid weakened the intensity of lactic fermentation and lowered the ratio of lactic to total organic acids in silages prepared with dried corn stover and cabbage waste. Interestingly, it was observed that adding 1,2-propanediol and 1-propanol also increased the pH, acetic acid and ethanol concentrations in the ensiled TMR. The effects of 1,2-propanediol and 1-propanol addition on fermentation have been rarely reported in the literature. However, Mukdsi et al [16] reported that some *lactobacilli* are capable of synthesizing esters with lactic acid and alcohol as the precursors. A possible explanation might be esterification of lactic acid and the alcohol which slowed the pH decline and increased the activities of acetic acid and ethanol-producing microorganisms, such as enterobacteria and yeasts, at early stages of ensiling. NH_3_-N is a sensitive indicator for silage proteolysis and its production is closely related to the pH decline during ensiling [17]. Higher NH_3_-N concentrations demonstrated the slower pH decline in 1,2-propanediol and 1-propanol-treated TMR in comparison to control.

Krooneman et al [18] previously isolated two strains of *L. diolivorans* from corn silage and demonstrated their abilities of fermenting 1,2-propanediol to propionic acid and 1-propanol. As propionic acid exhibits antimicrobial activity, this fermentative process also contributes to the improvement in aerobic stability of *L. buchneri*-inoculated silage [4]. In the experiment, adding 1,2-propanediol did not result in increases in propionic acid and 1-propanol concentrations suggesting the absence of *L. diolivorans* in the TMR. In addition, propionic acid addition did not elicit a significant effect on the suppression of aerobic bacteria and yeast when compared with control in this study (Table 3). This might be linked to the presence of microorganism species resistant to low concentrates of propionic acid. Crawshaw et al [19] found some yeasts still flourished in grass silage when treated with propionic acid at a level of 6 litres/t FW (equals to 8 g/kg DM).

### Additives on chemical composition and *in vitro* degradability

During ensiling process DM and nutrient losses are unavoidable and mainly results from plant respiration and activities of microorganisms [2]. Addition of 1,2-propanediol and 1-propanediol did not efficiently decline pH and resulted in lack of preventing microbial activity. This probably caused higher DM loss relative to other treatments (Table 4).

*In vitro* GP is often used as an important indicator for rumen digestibility potential [11]. In the present study, despite greater DM loss of 1,2-propanediol or 1-propanol treated TMR than others, *in vitro* digestibility parameters among the silages did not differ, suggesting none of these additives substantially affected ruminal digestion of the TMR.

### Additives on aerobic stability

Aerobic deterioration is initiated, in most cases, by acid-tolerant yeasts [20]. Yeasts can oxidize the fermentation products, leading to pH rises and proliferation of aerobic microorganisms in air-exposed silage. Aerobic deterioration of silage does not only cause losses of nutritional value, it also negatively affects the safety of silage with an increased risk of pathogenic microorganisms [21]. Well-fermented silages are easily subject to aerobic spoilage when exposed to aerobic condition because of degradation of lactic acid by yeasts and aerobic microbes [22,23]. It is generally believed that silages are prone to deteriorate when the yeast population exceeds 5.00 log_10_ cfu/g FW [24]. In the experiment, control began to spoil after 6 days and propionic acid-treated TMR became unstable after 14 days of aerobic exposure (Table 6). This may be linked with high yeast numbers (>5.00 log_10_ cfu/g FW) and accumulations of lactic acid (>72 g/kg DM) at the silos opening (Table 3). The TMRs treated with *L. buchneri*, acetic acid, 1,2-propanediol, and 1-propanol kept stable throughout the aerobic stability test (Table 6). 1,2-Propanediol itself has no inhibitory effects on yeast growth in silage, and 1-propanol only causes weak inhibition at concentrations of more than 20.0 g/kg DM [25]. While acetic acid is well known as an active substance for suppressing the proliferation of yeast, mold and fungi during aerobic exposure. This acid is typically produced in greater quantities than propionic acid during ensiling due to the hetero-fermentative metabolism of LAB [26]. High accumulations of acetic acid in these TMRs may be directly responsible for the increased aerobic stability. Furthermore, ethanol also exhibits antimicrobial activity and reportedly has the ability to potentiate the effect of acetic acid with respect to inhibition of fermenting yeasts [27]. High ethanol concentrations were also supposed to play an important role in preventing aerobic deterioration.

## CONCLUSION

The results of this study showed that treatments of *L. buchneri*, acetic acid, 1,2-propanediol, and 1-propanol did not alter *in vitro* digestibility, whereas they successfully modulated fermentation patterns towards producing more acetic acid and ethanol and substantially improved the aerobic stability of ensiled TMR. In addition, our findings also suggested that mechanisms of 1,2-propanediol and 1-propanol in altering fermentation may be different from that of *L. buchneri* or acetic acid. Further study is required to evaluate their effects on metabolic activities and microbial community structures during the ensiling.

## Figures and Tables

**Table 1:** Chemical composition of ingredients used for the formulation of total mixed ration

Items	Hulless barley straw	Tall fescue	Alfalfa	Wet hulless barley distillers’ grains	Concentrate
Dry matter (g/kg FW)	748	306	269	144	873
Crude protein (g/kg DM)	42.1	72.9	202	291	154
Water-soluble carbohydrates (g/kg DM)	44.8	144	85.0	56.6	103
Neutral detergent fiber (g/kg DM)	720	547	358	381	340
Acid detergent fiber (g/kg DM)	457	248	260	106	151
Ash (g/kg DM)	63.2	90.8	83.9	92.1	106
Ether extract (g/kg DM)	53.9	69.7	66.8	136	59.0
Buffering capacity (mEq/kg DM)	44.0	205	368	126	171

FW, fresh weight; DM, dry matter; mEq, milligram equivalent.

**Table 2:** Ingredients and chemical composition of total mixed rations without additives before ensiling

Items	Total mixed ration
Ingredients of feedstuff (g/kg DM)	
Hulless barley straw	160
Tall fescue	60
Alfalfa	170
Wet hulless barley distillers’ grains	60
Concentrate	550
Chemical composition	
DM (g/kg FW)	485
Crude protein (g/kg DM)	147
Water-soluble carbohydrates (g/kg DM)	90.5
Neutral detergent fiber (g/kg DM)	418
Acid detergent fiber (g/kg DM)	221
Ash (g/kg DM)	93.9
Ether extract (g/kg DM)	64.8
Buffering capacity (mEq/kg DM)	184
Microbial composition	
Lactic acid bacteria (log_10_ cfu/g FW)	5.37
Aerobic bacteria (log_10_ cfu/g FW)	6.44
Yeast (log_10_ cfu/g FW)	5.21

DM, dry matter; FW, fresh weight; mEq, milligram equivalent; cfu, colony-forming units.

**Table 3:** Fermentation parameters and microbial compositions of total mixed ration after 90 days of ensiling

Items	Treatments1)						SEM	p-value

	Control	B	A	P	E	F		
Fermentation parameters								
pH	4.27^A^	4.53^BC^	4.45^B^	4.35^A^	4.59^C^	4.58^C^	0.013	<0.001
Lactic acid (g/kg DM)	74.3^B^	65.6^AB^	67.8^AB^	72.6^B^	62.0^A^	60.0^A^	1.073	0.013
Acetic acid (g/kg DM)	21.0^A^	38.0^B^	37.0^B^	21.0^A^	34.5^B^	40.1^B^	0.528	<0.001
Lactic acid/acetic acid	3.54^B^	1.73^A^	1.83^A^	3.46^B^	1.80^A^	1.50^A^	0.069	<0.001
Propionic acid (g/kg DM)	6.30^D^	1.50^B^	0.00^A^	7.40^D^	4.19^C^	3.10^C^	0.112	<0.001
Ethanol (g/kg DM)	22.4^A^	41.8ABC	42.86ABC	29.6^A^	55.2^BC^	59.2^C^	3.796	0.009
1,2-propanediol (g/kg DM)	0.00^A^	0.00^A^	0.00^A^	0.00^A^	5.45^B^	0.12^A^	0.008	<0.001
1-propanol (g/kg DM)	0.00^A^	0.00^A^	0.00^A^	0.00^A^	0.00^A^	4.87^B^	0.005	0.012
Butyric acid (g/kg DM)	0.00	0.00	0.00	0.00	0.00	0.00	0.00	-
Ammonia nitrogen (g/kg TN)	76.7^AB^	81.7ABC	74.4^AB^	70.7^A^	90.1^C^	85.3^BC^	2.094	0.040
Microbial compositions								
Lactic acid bacteria	7.53	6.86	6.83	7.52	7.6	7.34	0.115	0.256
Aerobic bacteria	6.66^B^	6.07^B^	5.17^A^	6.78^B^	6.79^B^	6.61^B^	0.077	<0.001
Yeast	5.55^C^	1.40^AB^	1.77^AB^	5.55^C^	2.94^BC^	0.00^A^	0.354	0.003

SEM, standard error of means; DM, dry matter; TN, total nitrogen.

1) B, *L. buchneri*; A, acetic acid; P, propionic acid; E, 1,2-propanediol; F, 1-propanol.

A–D Means in the same row with different superscripts differ (p<0.05).

**Table 4:** Chemical compositions of the total mixed ration after 90 days of ensiling

Items	Treatments1)						SEM	p-value

	Control	B	A	P	E	F		
Dry matter (g/kg FW)	477^A^	466^B^	480^A^	482^A^	451^B^	463^B^	3.386	0.019
Dry matter loss	4.02^A^	6.24^B^	3.42^A^	3.02^A^	9.26^B^	6.84^B^	0.012	0.023
Crude protein (g/kg DM)	159	155	154	168	160	156	1.649	0.234
Water-soluble carbohydrates (g/kg DM)	16.5	18.3	17.7	19.5	13.5	15.8	1.012	0.627
Neutral detergent fiber (g/kg DM)	412	408	386	405	441	397	9.878	0.765
Acid detergent fiber (g/kg DM)	182	212	225	233	240	230	11.381	0.792
Ash (g/kg DM)	87.8	91.8	102	97.9	123	105	3.831	0.097
Ether extract (g/kg DM)	76.5	73.9	80.6	85.4	91.1	89.4	2.045	0.068

SEM, standard error of means; FW, fresh weight; DM, dry matter.

1) B, *L. buchneri*; A, acetic acid; P, propionic acid; E, 1,2-propanediol; F, 1-propanol.

AB Means in the same row with different superscripts differ (p<0.05).

**Table 5:** *In vitro* degradability and gas production kinetics of total mixed ration after 90 days of ensiling

Items	Treatments1)						SEM	p-value

	Control	B	A	P	E	F		
Degradability								
IVDMD	269	297	271	294	305	315	5.939	0.123
IVNDFD	145	147	146	152	167	169	2.413	0.064
*In vitro* gas production kinetics								
GP_24_ (mL)	110	96.4	111	110	123	126	6.292	0.066
Rate of GP (mL/h)	0.047	0.039	0.051	0.058	0.047	0.042	0.003	0.210
Potential GP (mL)	173	175	162	158	161	200	6.943	0.137

SEM, standard error of means; DM, dry matter; IVDMD, *in vitro* degradability of DM; IVNDFD, *in vitro* degradability of neutral detergent fibre; GP_24_, gas production at 24 h.

1) B, *L. buchneri*; A, acetic acid; P, propionic acid; E, 1,2-propanediol; F, 1-propanol.

**Table 6:** Changes in fermentative characteristics of fermented total mixed ration during aerobic exposure

Items	Treatments1)	Days of exposure (d)					SEM	p-value2)		
	
		0	3	6	9	14		T	D	T×D
pH	Control	4.27^A^^a^	4.36^A^^ab^	4.39^A^^b^	4.87^C^^c^	5.95^C^^d^	0.008	<0.001	<0.001	<0.001
	B	4.53^C^	4.56^B^	4.56^B^	4.58^BC^	4.58^AB^				
	A	4.45^BC^	4.41^A^	4.42^A^	4.45^A^	4.45^A^				
	P	4.35^AB^^a^	4.36^A^^a^	4.36^A^^a^	4.49^A^^b^	5.16^B^^c^				
	E	4.59^C^	4.64^C^	4.61^B^	4.64^BC^	4.62^AB^				
	F	4.58^C^	4.60^BC^	4.57^B^	4.62^BC^	4.60^AB^				
Lactic acid (g/kg DM)	Control	74.3^B^^c^	90.0^B^^c^	36.4^A^^b^	38.5^A^^b^	25.0^A^^a^	0.914	<0.001	<0.001	0.001
	B	65.6^AB^^b^	69.0^A^^b^	45.9^B^^a^	56.1^B^^ab^	55.8^B^^ab^				
	A	67.8^AB^^b^	72.3^BC^^b^	42.6^B^^a^	79.2^C^^b^	61.5^B^^ab^				
	P	72.6^B^^c^	77.6^BC^^c^	52.5^B^^b^	38.5^A^^ab^	30.2^A^^a^				
	E	62.2^AB^^b^	58.8^A^^ab^	48.8^B^^a^	49.0^B^^a^	59.4^B^^ab^				
	F	59.9^A^^ab^	61.7^A^^ab^	62.1^C^^b^	52.4^B^^a^	53.0^B^^a^				
Acetic acid (g/kg DM)	Control	20.6^A^^b^	21.8^AB^^b^	12.0^A^^ab^	11.8^A^^ab^	4.85^A^^a^	0.449	<0.001	<0.001	<0.001
	B	38.1^B^^c^	29.2^B^^b^	20.3^BC^^a^	32.2^B^^b^	31.0^B^^b^				
	A	37.1^B^	31.8^B^	21.9^C^	39.0^B^	31.9^B^				
	P	20.7^A^	16.2^A^	12.8^AB^	21.0^AB^	9.95^A^				
	E	34.5^B^^b^	26.2^AB^^a^	27.1^C^^a^	29.4^AB^^ab^	28.7^B^^a^				
	F	40.1^B^	32.6^B^	41.9^D^	36.1^B^	34.3^B^				
Propionic acid (g/kg DM)	Control	6.27^D^^c^	0.03^A^^a^	2.50^A^^b^	2.85^A^^b^	2.48^A^^b^	0.078	<0.001	<0.001	<0.001
	B	1.53^B^^a^	1.22^AB^^a^	2.99^A^^b^	3.72^A^^c^	3.81^AB^^c^				
	A	0.00^A^^a^	0.01^A^^a^	2.85^A^^b^	3.81^A^^c^	3.43^AB^^bc^				
	P	7.36^D^^ab^	5.57^C^^a^	5.02^B^^a^	9.22^B^^c^	7.99^D^^b^				
	E	4.19^C^^ab^	2.67^B^^a^	5.27^B^^b^	5.46^A^^b^	5.56^C^^b^				
	F	3.11^C^^a^	2.55^B^^a^	5.04^B^^b^	4.47^A^^b^	4.46^BC^^b^				
Ethanol (g/kg DM)	Control	22.4^A^	11.1^A^	6.08^A^	1.03^A^	0.00^A^	0.614	<0.001	<0.001	0.068
	B	41.8^AB^^c^	15.1^AB^^ab^	12.6^AB^^a^	18.5^BC^^b^	11.5^B^^a^				
	A	42.9^AB^^b^	15.8^AB^^a^	13.4^B^^a^	21.7^BC^^a^	15.9^B^^a^				
	P	29.6^A^^b^	10.6^A^^a^	8.72^AB^^a^	8.47^AB^^a^	2.11^A^^a^				
	E	55.2^C^^b^	18.2^AB^^a^	20.3^C^^a^	26.4^B^^a^	25.5^C^^a^				
	F	59.2^C^^d^	21.7^B^^a^	32.7^D^^c^	29.8^B^^bc^	26.0^C^^ab^				

SEM, standard error of means; DM, dry matter.

1) B, *L. buchneri*; A, acetic acid; P, propionic acid; E,1,2-propanediol; F, 1-propanol.

2) T, treatment; D, ensiling day; T×D, interaction between treatment and ensiling day.

A–D Means in the same column with different capital letter differed (p<0.05).

a–d Means in the same row with different lowercase differed (p<0.05).

**Table 7:** Changes in chemical compositions of fermented total mixed ration during aerobic exposure

Items	Treatments1)	Days of exposure (d)					SEM	p-value2)		
	
		0	3	6	9	14		T	D	T×D
DM (g/kg FW)	Control	477^A^^a^	524^b^	531^b^	538^b^	496^a^	1.102	0.004	<0.001	0.137
	B	466^B^^a^	531^c^	520^bc^	530^c^	506^b^				
	A	480^A^^a^	525^bc^	526^bc^	544^c^	501^ab^				
	P	482^A^^a^	535^ab^	534^ab^	538^c^	509^ab^				
	E	451^B^^a^	529^c^	530^c^	521^bc^	489^b^				
	F	463^B^^a^	532^b^	532^b^	537^b^	483^a^				
Water-soluble carbohydrates (g/kg DM)	Control	16.5^a^	16.3^a^	26.8^b^	15.0^a^	12.1^A^^a^	0.451	0.005	0.001	0.209
	B	18.3	15.7	24.2	21.1	16.6^AB^				
	A	17.7	21.6	27.1	19.5	16.9^AB^				
	P	19.5	22.0	28.4	26.2	21.1^AB^				
	E	13.5^a^	16.0^a^	29.5^b^	16.7^a^	18.9^AB^^ab^				
	F	15.8	22.6	22.3	23.6	23.1^B^				
Ammonia nitrogen (g/kg TN)	Control	76.7^AB^	70.6	68.4	69.4	63.2^A^	1.314	<0.001	<0.001	0.017
	B	81.7^AB^^ab^	59.2^a^	66.6^a^	53.5^a^	99.0^BC^^b^				
	A	74.4^AB^	72.1	72.4	84.4	99.3^BC^				
	P	70.7^A^^ab^	61.4^a^	58.5^a^	61.3^a^	76.3^AB^^b^				
	E	90.1^B^^bc^	72.2abc	66.4^ab^	63.8^a^	95.1^BC^^c^				
	F	85.3^AB^	70.9	65.8	101	114^C^				

SEM, standard error of means; DM, dry matter; FW, fresh weight; TN, total nitrogen.

1) B, *L. buchneri*; A, acetic acid; P, propionic acid; E, 1,2-propanediol; F,1-propanol.

2) T, treatment; D, ensiling day; T×D, interaction between treatment and ensiling day.

A–C Means in the same column with different capital letter differed (p<0.05).

a–c Means in the same row with different lowercase differed (p<0.05).

**Table 8:** Changes in microbial composition of fermented total mixed ration during aerobic exposure

Items	Treatments1)	Days of exposure (d)					SEM	p-value2)		
	
		0	3	6	9	14		T	D	T×D
Lactic acid bacteria (log_10_ cfu/g FW)	Control	7.53^c^	7.05^a^	7.30abc	7.38^bc^	7.17^ab^	0.035	0.042	0.016	0.110
	B	6.86	7.02	7.41	7.26	7.12				
	A	6.83	6.90	7.26	6.64	7.11				
	P	7.52	6.94	7.34	7.43	7.42				
	E	7.60	7.30	7.45	6.88	6.66				
	F	7.34	7.11	7.40	6.89	6.93				
Aerobic bacteria (log_10_ cfu/g FW)	Control	6.66^B^^a^	6.77^B^^a^	6.52^B^^a^	6.32^B^^a^	7.57^B^^b^	0.003	<0.001	<0.001	<0.001
	B	6.07^B^^ab^	5.68^AB^^a^	5.70^AB^^a^	5.62^AB^^a^	6.79^AB^^b^				
	A	5.17^A^^a^	5.20^A^^a^	5.72^A^^b^	5.47^AB^^ab^	6.82^AB^^c^				
	P	6.78^B^^a^	6.96^B^^a^	6.89^B^^a^	6.23^B^^a^	7.39^B^^b^				
	E	6.79^B^^b^	5.23^A^^a^	6.00^AB^^ab^	5.20^A^^a^	6.49^AB^^b^				
	F	6.61^B^^c^	5.64^A^^a^	6.09^AB^^ab^	5.79^AB^^a^	6.14^A^^b^				
Yeast (log_10_ cfu//kg FW)	Control	5.50^BC^^a^	5.61^B^^ab^	5.93^C^abc	5.99^C^^bc^	6.13^B^^c^	0.160	<0.001	<0.001	0.003
	B	1.40^AB^^ab^	1.40^AB^^ab^	1.20^AB^^ab^	0.00^A^^a^	4.92^AB^^b^				
	A	1.77^AB^	0.00^A^	2.60ABC	1.53^AB^	3.05^A^				
	P	5.55^C^	5.10^B^	4.97^BC^	5.90^C^	6.10^B^				
	E	2.94ABC	1.48^AB^	1.36^AB^	0.00^A^	3.19^A^				
	F	0.00^A^^a^	1.20^AB^^a^	0.00^A^^a^	4.42^BC^^b^	4.53^AB^^b^				

SEM, standard error of means; FW, fresh weight; cfu, colony-forming units.

1) B, *L. buchneri*; A, acetic acid; P, propionic acid; E, 1,2-propanediol; F, 1-propanol.

2) T, treatment; D, ensiling day; T×D, interaction between treatment and ensiling day.

A–C Means in the same column with different capital letter differed (p<0.05).

a–c Means in the same row with different lowercase differed (p<0.05).

## References

[1] Kleinschmit DH, Kung L (2006). A meta-analysis of the effects of *Lactobacillus buchneri* on the fermentation and aerobic stability of corn and grass and small-grain silages. J Dairy Sci.

[2] Dong Z, Yuan X, Wen A, Desta ST, Shao T (2017). Effects of calcium propionate on the fermentation quality and aerobic stability of alfalfa silage. Asian-Australas J Anim.

[3] Li Y, Nishino N (2011). Effects of inoculation of *Lactobacillus rhamnosus* and *Lactobacillus buchneri* on fermentation, aerobic stability and microbial communities in whole crop corn silage. Grassl Sci.

[4] Nishino N, Yoshida M, Shiota H, Sakaguchi E (2003). Accumulation of 1,2-propanediol and enhancement of aerobic stability in whole crop maize silage inoculated with *Lactobacillus buchneri*. J Appl Microbiol.

[5] Auesukaree C, Damnernsawad A, Kruatrachue M (2009). Genome-wide identification of genes involved in tolerance to various environmental stresses in *Saccharomyces cerevisiae*. J Appl Genet.

[6] Carvalho BF, Ávila CLS, Pinto JC, Pereira MN, Schwan RF (2012). Effects of propionic acid and *Lactobacillus buchneri* (UFLA SIL 72) addition on fermentative and microbiological characteristics of sugar cane silage treated with and without calcium oxide. Grass Forage Sci.

[7] Latimer GW (2019). AOAC International Official methods of analysis.

[8] Van Soest PJ, Robertson JB, Lewis BA (1991). Methods for dietary fiber, neutral detergent fiber, and nonstarch polysaccharides in relation to animal nutrition. J Dairy Sci.

[9] Broderick GA, Kang JH (1980). Automated simultaneous determination of ammonia and total amino acids in ruminal fluid and *in vitro* media. J Dairy Sci.

[10] Chen L, Yuan XJ, Li JF, Wang SR, Dong ZH, Shao T (2017). Effect of lactic acid bacteria and propionic acid on conservation characteristics, aerobic stability and *in vitro* gas production kinetics and digestibility of whole-crop corn based total mixed ration silage. J Integr Agric.

[11] Contreras-Govea FE, Muck RE, Mertens DR, Weimer PJ (2011). Microbial inoculant effects on silage and *in vitro* ruminal fermentation, and microbial biomass estimation for alfalfa, bmr corn, and corn silages. Anim Feed Sci Technol.

[12] Liu Q, Zhang J, Shi S, Sun Q (2011). The effects of wilting and storage temperatures on the fermentation quality and aerobic stability of stylo silage. Anim Sci J.

[13] Wang Y, Wang C, Zhou W, Yang FY, Chen XY, Zhang Q (2018). Effects of wilting and *Lactobacillus plantarum* addition on the fermentation quality and microbial community of Moringa oleifera leaf silage. Front Microbiol.

[14] McDonald P, Henderson AR, Heron SJE (1991). The biochemistry of silage.

[15] Ren H, Wang C, Fan W, Zhang B, Li Z, Li D (2018). Effects of formic or acetic acid on the storage quality of mixed air-dried corn stover and cabbage waste, and microbial community analysis. Food Technol Biotechnol.

[16] Mukdsi MCA, Maillard MB, Medina RB, Thierry A (2018). Ethyl butanoate is synthesised both by alcoholysis and esterification by dairy lactobacilli and propionibacteria. LWT.

[17] Guo X, Zhou H, Yu Z, Zhang Y (2007). Changes in the distribution of nitrogen and plant enzymatic activity during ensilage of lucerne treated with different additives. Grass Forage Sci.

[18] Krooneman J, Faber F, Alderkamp AC (2002). *Lactobacillus diolivorans* sp. nov., a 1,2-propanediol-degrading bacterium isolated from aerobically stable maize silage. Int J Syst Evol Microbiol.

[19] Crawshaw R, Thorne DM, Llewelyn RH (1980). The effects of formic and propionic acids on the aerobic deterioration of grass silage in laboratory units. J Sci Food Agric.

[20] Bernardes TF, De Oliveira IL, Lara MAS, Casagrande DR, Ávila CLS, Pereira OG (2015). Effects of potassium sorbate and sodium benzoate at two application rates on fermentation and aerobic stability of maize silage. Grass Forage Sci.

[21] Bayatkouhsar J, Tahmasbi AM, Naserian AA (2012). Effects of microbial inoculant on composition, aerobic stability, *in situ* ruminal degradability and *in vitro* gas production of corn silage. Int J Agrisci.

[22] Nishino N, Touno E (2005). Ensiling characteristics and aerobic stability of direct-cut and wilted grass silages inoculated with *Lactobacillus casei* or *Lactobacillus buchneri*. J Sci Food Agric.

[23] Kung L, Ranjit NK (2001). The effect of *Lactobacillus buchneri* and other additives on the fermentation and aerobic stability of barley silage. J Dairy Sci.

[24] Filya I, Sucu E (2010). The effects of lactic acid bacteria on the fermentation, aerobic stability and nutritive value of maize silage. Grass Forage Sci.

[25] Danner H, Holzer M, Mayrhuber E, Braun R (2003). Acetic acid increases stability of silage under aerobic conditions. Appl Environ Microbiol.

[26] Kung L, Shaver RD, Grant RJ, Schmidt RJ (2018). Silage review: interpretation of chemical, microbial, and organoleptic components of silages. J Dairy Sci.

[27] Pampulha ME, Loureiro-Dias MC (1989). Combined effect of acetic acid, pH and ethanol on intracellular pH of fermenting yeast. Appl Microbiol Biotechnol.

